# Symptoms of Depression, Anxiety, Post-Traumatic Stress Disorder, and Suicidal Ideation Among State, Tribal, Local, and Territorial Public Health Workers During the COVID-19 Pandemic — United States, March–April 2021

**DOI:** 10.15585/mmwr.mm7048a6

**Published:** 2021-12-03

**Authors:** Jonathan Bryant-Genevier, Carol Y. Rao, Barbara Lopes-Cardozo, Ahoua Kone, Charles Rose, Isabel Thomas, Diana Orquiola, Ruth Lynfield, Dhara Shah, Lori Freeman, Scott Becker, Amber Williams, Deborah W. Gould, Hope Tiesman, Geremy Lloyd, Laura Hill, Ramona Byrkit

**Affiliations:** ^1^Epidemic Intelligence Service, CDC; ^2^CDC COVID-19 Response Team; ^3^Minnesota Department of Health; ^4^Council of State and Territorial Epidemiologists, Atlanta, Georgia; ^5^National Association of County and City Health Officials, Washington, DC; ^6^Association of Public Health Laboratories, Silver Spring, Maryland; ^7^Association of State and Territorial Health Officials, Arlington, Virginia.

Increases in mental health conditions have been documented among the general population and health care workers since the start of the COVID-19 pandemic (*1*–*3*). Public health workers might be at similar risk for negative mental health consequences because of the prolonged demand for responding to the pandemic and for implementing an unprecedented vaccination campaign. The extent of mental health conditions among public health workers during the COVID-19 pandemic, however, is uncertain. A 2014 survey estimated that there were nearly 250,000 state and local public health workers in the United States (*4*). To evaluate mental health conditions among these workers, a nonprobability–based online survey was conducted during March 29–April 16, 2021, to assess symptoms of depression, anxiety, post-traumatic stress disorder (PTSD), and suicidal ideation among public health workers in state, tribal, local, and territorial public health departments. Among 26,174 respondents, 52.8% reported symptoms of at least one mental health condition in the preceding 2 weeks, including depression (30.8%), anxiety (30.3%), PTSD (36.8%), or suicidal ideation (8.4%). The highest prevalence of symptoms of a mental health condition was among respondents aged ≤29 years (range = 13.6%–47.4%) and transgender or nonbinary persons (i.e., those who identified as neither male nor female) of all ages (range = 30.4%–65.5%). Public health workers who reported being unable to take time off from work were more likely to report adverse mental health symptoms. Severity of symptoms increased with increasing weekly work hours and percentage of work time dedicated to COVID-19 response activities. Implementing prevention and control practices that eliminate, reduce, and manage factors that cause or contribute to public health workers’ poor mental health might improve mental health outcomes during emergencies.

A nonprobability–based convenience sample of public health workers was invited to complete a self-administered, online, anonymous survey during March 29–April 16, 2021. All persons who worked at a state, tribal, local, or territorial health department for any length of time in 2020 were eligible to participate.* National public health membership associations^†^ emailed a link to the survey to all members (approximately 24,000), and supervisors were asked to cascade the survey to all workers within their organization; 26,174 public health workers responded to the survey. The survey included questions on traumatic events or stressors experienced since March 2020,^§^ demographics, workplace factors, and self-reported mental health symptoms, including depression, anxiety, PTSD, or suicidal ideation, in the past 2 weeks. Mental health symptoms were evaluated using the 9-item Patient Health Questionnaire (PHQ-9) for depression (*5*), the 2-item General Anxiety Disorder (GAD-2) for anxiety (*6*), the 6-item Impact of Event Scale (IES-6) for PTSD (*7*),^¶^ and one item of the PHQ-9 for suicidal ideation.** Prevalence of symptoms of mental health conditions and suicidal ideation were assessed by demographic characteristics and workplace factors.^††^ Univariate prevalence ratios were calculated using Poisson regression with 95% confidence intervals estimated using a robust standard error. Analyses were completed using RStudio software (version 1.2.1335; RStudio). This activity was reviewed by CDC and was conducted consistent with applicable federal law and CDC policy.^§§^

Overall, 52.8% of respondents reported symptoms of at least one adverse mental health condition in the preceding 2 weeks. Prevalences of symptoms of depression, anxiety, PTSD, and suicidal ideation were 30.8%, 30.3%, 36.8%, and 8.4%, respectively (Table 1). The highest prevalences of symptoms of a mental health condition or suicidal ideation were among respondents aged ≤29 years (range = 13.6%–47.4%), transgender or nonbinary persons of all ages (range = 30.4%–65.5%), and those who identified as multiple races (range = 12.1%–43.4%); prevalence of symptoms of PTSD was higher among respondents who had a postbaccalaureate graduate education (40.7%).

**TABLE 1:** Mental health symptoms among 26,174 state, tribal, local, and territorial public health workers during the past 2 weeks, by demographic characteristics and work factors — United States, March–April 2021

Characteristic	No.	Depression* (n = 22,692^†^)		Anxiety* (n = 23,610^†^)		PTSD* (n = 22,248^†^)		Suicidal ideation (n = 23,317^†^)	
		Prevalence, %	PR (95% CI)	Prevalence, %	PR (95% CI)	Prevalence, %	PR (95% CI)	Prevalence, %	PR (95% CI)
**Overall**	26,174*	30.8	––	30.3	––	36.8	––	8.4	––
**Age group, yrs**									
≤29	3,525	40.3	2.11 (1.93–2.30)	44.7	2.81 (2.56–3.09)	47.4	2.03 (1.88–2.19)	13.6	2.98 (2.46–3.60)
30–39	5,461	34.3	1.80 (1.65–1.96)	37.1	2.33 (2.12–2.56)	42.3	1.81 (1.68–1.95)	10.3	2.26 (1.87–2.73)
40–49	5,102	31.4	1.64 (1.50–1.80)	29.1	1.83 (1.66–2.01)	37.3	1.60 (1.48–1.73)	7.5	1.65 (1.36–2.01)
50–59	4,925	27.6	1.45 (1.32–1.58)	23.5	1.47 (1.33–1.63)	32.0	1.37 (1.26–1.48)	6.0	1.32 (1.08–1.62)
≥60	2,830	19.1	Ref	15.9	Ref	23.4	Ref	4.6	Ref
**Sex**									
Male	3,904	27.1	Ref	24.4	Ref	33.2	Ref	9.9	Ref
Female	19,873	31.2	1.15 (1.09–1.22)	31.2	1.28 (1.20–1.36)	37.2	1.12 (1.07–1.18)	7.9	0.81 (0.72–0.90)
Transgender or nonbinary	147	61.9	2.29 (1.98–2.64)	61.1	2.21 (1.88–2.59)	65.5	1.97 (1.74–2.24)	30.4	3.10 (2.37–4.06)
**Race/Ethnicity**									
Hispanic	1,974	30.0	0.95 (0.89–1.03)	29.9	0.95 (0.89–1.02)	37.5	1.01 (0.95–1.07)	9.9	1.20 (1.03–1.39)
AI/AN, NH	156	35.8	1.14 (0.92–1.41)	32.7	1.04 (0.83–1.31)	41.6	1.12 (0.92–1.35)	7.3	0.89 (0.50–1.57)
Asian, NH	1,009	28.3	0.90 (0.81–1.00)	27.6	0.88 (0.79–0.98)	38.3	1.03 (0.94–1.12)	10.1	1.22 (1.00–1.49)
Black, NH	2,177	24.4	0.77 (0.71–0.84)	21.7	0.69 (0.64–0.75)	29.8	0.80 (0.75–0.86)	6.5	0.79 (0.67–0.94)
NH/PI, NH	96	26.5	0.84 (0.59–1.21)	22.2	0.71 (0.48–1.04)	25.3	0.68 (0.47–0.98)	11.1	1.34 (0.75–2.42)
White, NH	17,218	31.5	Ref	31.4	Ref	37.2	Ref	8.3	Ref
Multiple races, NH	614	39.6	1.26 (1.14–1.39)	37.2	1.19 (1.07–1.32)	43.4	1.17 (1.06–1.28)	12.1	1.46 (1.17–1.83)
**Highest educational degree attained**									
Less than bachelor’s	5,386	31.0	Ref	27.1	Ref	30.1	Ref	6.5	Ref
Bachelor’s	9,180	31.4	1.01 (0.96–1.07)	30.6	1.13 (1.07–1.20)	36.8	1.22 (1.16–1.29)	9.1	1.40 (1.24–1.59)
Graduate	9,375	30.4	0.98 (0.93–1.04)	32.0	1.18 (1.12–1.25)	40.7	1.35 (1.29–1.42)	8.9	1.37 (1.22–1.56)
**Hrs worked per wk**									
≤40	9,993	23.5	Ref	24.4	Ref	27.3	Ref	7.6	Ref
41–60	11,466	33.3	1.42 (1.35–1.48)	32.3	1.32 (1.26–1.38)	40.4	1.48 (1.42–1.54)	8.4	1.10 (1.00–1.21)
>60	3,018	45.6	1.94 (1.84–2.05)	41.6	1.70 (1.61–1.80)	54.2	1.99 (1.89–2.08)	11.0	1.44 (1.27–1.63)
**% of time spent on COVID–19 response activities**									
None	1,787	22.5	Ref	23.0	Ref	22.3	Ref	7.6	Ref
1–25	5,151	23.6	1.05 (0.95–1.17)	23.5	1.02 (0.92–1.13)	24.3	1.09 (0.98–1.21)	7.5	0.99 (0.82–1.21)
26–50	3,432	27.6	1.23 (1.11–1.37)	26.7	1.16 (1.05–1.29)	31.6	1.42 (1.28–1.57)	8.4	1.12 (0.91–1.37)
51–75	3,283	30.6	1.36 (1.23–1.51)	30.6	1.33 (1.20–1.47)	37.0	1.66 (1.50–1.84)	8.6	1.14 (0.93–1.40)
≥76	10,620	36.9	1.64 (1.50–1.81)	35.9	1.56 (1.42–1.71)	47.0	2.11 (1.92–2.32)	8.9	1.18 (0.99–1.41)
**Can take time off from work**									
Yes	13,507	22.6	Ref	23.0	Ref	27.9	Ref	6.2	Ref
No	8,586	44.1	1.95 (1.87–2.03)	42.4	1.85 (1.77–1.92)	51.5	1.84 (1.78–1.91)	12.0	1.92 (1.76–2.10)

**Abbreviations:** AI/AN = American Indian or Alaska Native; CI = confidence interval; IES-6 = 6-item Impact of Event Scale; GAD-2 = General Anxiety Disorder; NH = non-Hispanic; NH/PI = Native Hawaiian or Pacific Islander; PHQ-9 = 9-item Patient Health Questionnaire; PR = prevalence ratio; PTSD = post-traumatic stress disorder; Ref = referent group.

* Symptoms of mental health conditions were scored and categorized by severity. Respondents who scored ≥10.0 out of 27 on the PHQ-9 for depression, ≥3.0 out of 6 on the GAD-2 for anxiety, or ≥1.75 out of 4 on the IES-6 for PTSD were considered symptomatic for the respective conditions. Respondents who indicated that they would be better off dead or thought of hurting themselves at any time in the past 2 weeks were categorized as experiencing suicidal ideation.

^†^ Some categories might not sum to 26,174 because of missing data. Denominators for categories are respondents who answered the questions to be scored.

Most (92.6%) respondents reported working directly on COVID-19 response activities; the majority (59.2%) worked ≥41 hours in a typical week since March 2020. The prevalences of all four mental health outcomes and the severity of symptoms of depression or PTSD increased as the percentage of work time spent directly on COVID-19 response activities and number of work hours in a typical week increased (Table 1) (Figure). Public health workers who were unable to take time off from work when they needed were nearly twice as likely to report symptoms of an adverse mental health condition (prevalence ratio range = 1.84–1.95) as were those who could take time off. Among those not able to take time off from work (8,586), the most common reasons were concern about falling behind on work (64.4%), no work coverage (60.6%), and feeling guilty (59.0%); 18.2% reported that their employer did not allow time off from work. Needing mental health counseling/services in the last 4 weeks, but not receiving these services, was reported by nearly one in five (19.6%) respondents. Employee assistance programs were available to nearly two thirds (66.1%) of respondents but were accessed by only 11.7% of those respondents; 27.3% of all respondents did not know whether their employer offered an employee assistance program.

**FIGURE:**
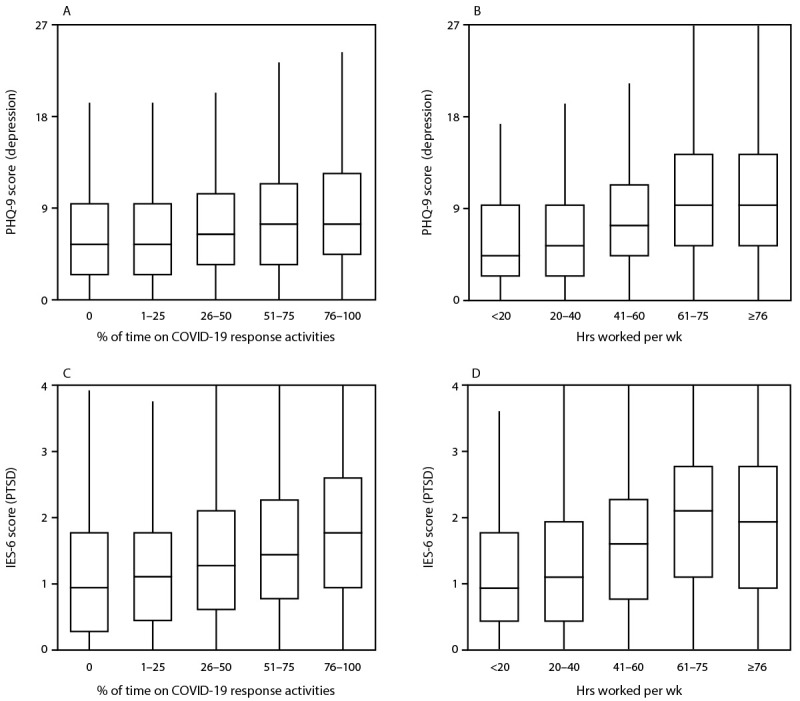
Distribution* of 9-item Patient Health Questionnaire scores for depression and 6-item Impact of Event Scale scores for post-traumatic stress disorder^†^ among state, tribal, local, and territorial public health worker respondents,^§^ by percentage of work time spent directly on COVID-19 response activities for the majority of 2020 (panels A, C), and hours worked in a typical week since March 2020 (panels B, D) — United States, March–April 2021 **Abbreviations:** IES-6 = 6-item Impact of Event Scale; PHQ-9 = 9-item Patient Health Questionnaire; PTSD = post-traumatic stress disorder. * Upper and lower levels of boxes indicate 75th and 25th percentiles, respectively; horizontal line indicates median; whiskers indicate observation nearest to 1.5 × interquartile range. ^†^ Self-reported symptoms of depression or PTSD were evaluated; respondents who scored ≥10.0 out of 27 on the PHQ-9 for depression or ≥1.75 out of 4 on the IES-6 for PTSD were considered symptomatic for the respective conditions. ^§^ Only public health worker respondents who completed all PHQ-9 items (n = 22,692) or all IES-6 items (n = 22,248) are included.

Respondents reported experiencing traumatic events or stressors since March 2020, including feeling overwhelmed by workload or family/work balance (72.0%), receiving job-related threats because of work (11.8%), and feeling bullied, threatened or harassed because of work (23.4%); 12.6% of respondents reported having received a diagnosis of COVID-19 (Table 2). Respondents who reported traumatic events or stressors, either personal or work-related, were more likely to report symptoms of PTSD than respondents who did not experience these events or stressors.

**TABLE 2:** Traumatic events or stressors reported by 26,174 state, tribal, local, and territorial public health workers and comparisons* of symptoms of post-traumatic stress disorder^†^ — United States, March–April 2021

Traumatic event or stressor/Response	No.^§^	PTSD prevalence, %	PTSD PR (95% CI)
**Personal-related**			
**Had COVID-19**			
Yes^¶^	2,834	36.7	1.03 (0.98–1.09)
Maybe**	3,310	42.4	1.19 (1.14–1.25)
No	16,266	35.6	Ref
**Got divorced or separated**			
Yes	747	49.6	1.36 (1.27–1.47)
No	22,084	36.3	Ref
**Experienced death of a loved one**			
Yes	7,580	42.3	1.24 (1.20–1.29)
No	15,403	34.0	Ref
**Worried about the health of family and loved ones**			
Yes	20,857	39.4	3.11 (2.77–3.48)
No	2,203	12.7	Ref
**Felt isolated and alone**			
Yes	12,944	49.8	2.49 (2.38–2.60)
No	10,080	20.0	Ref
**Work-related**			
**Felt overwhelmed by workload or family/work balance**			
Yes	16,563	45.4	3.10 (2.91–3.30)
No	6,451	14.7	Ref
**Felt disconnected from family and friends because of workload**			
Yes	14,051	49.0	2.77 (2.64–2.91)
No	8,964	17.7	Ref
**Felt inadequately compensated for work**			
Yes	13,703	45.2	1.85 (1.78–1.93)
No	9,101	24.4	Ref
**Felt unappreciated at work**			
Yes	12,362	46.5	1.82 (1.76–1.90)
No	10,551	25.5	Ref
**Experienced stigma or discrimination because of work**			
Yes	5,962	56.2	1.88 (1.82–1.94)
No	16,944	29.9	Ref
**Received job-related threats because of work**			
Yes	2,699	61.8	1.85 (1.78–1.92)
No	20,262	33.4	Ref
**Felt bullied, threatened, or harassed because of work**			
Yes	5,376	59.0	1.97 (1.91–2.03)
No	17,594	30.0	Ref
**Interacted often with the public**			
Yes	11,143	41.1	1.23 (1.19–1.28)
No	13,318	33.3	Ref
**Worried about workplace exposure to COVID-19**			
Yes	11,197	42.6	1.36 (1.31–1.41)
No	11,805	31.3	Ref

**Abbreviations:** IES-6 = 6-item Impact of Event Scale; PR = prevalence ratio; PTSD = post-traumatic stress disorder; Ref = referent group.

* Referent group for all prevalence ratio calculations was not experiencing the traumatic event/stressor (i.e., “No” category).

^†^ Experienced symptoms of post-traumatic stress disorder in the 2 weeks preceding survey, defined as having an IES-6 score ≥1.75 out of 4.

^§^ Some categories might not sum to 26,174; only those respondents who completed IES-6 questions (N = 22,248) are included in analysis.

^¶^ Positive COVID-19 test or diagnosis by medical professional.

** Had symptoms compatible with COVID-19 but not tested or test inconclusive.

## Discussion

Among a convenience sample of 26,174 state, tribal, local, and territorial public health workers, approximately one half experienced symptoms of a mental health condition in the 2 weeks preceding the survey, with highest prevalences reported among younger respondents, and transgender or nonbinary respondents. Public health workers who reported certain workplace practices, such as long work hours and the inability to take time off, were more likely to have experienced symptoms of a mental health condition. Implementing prevention and control practices that eliminate, reduce, and manage workplace factors^¶¶^ that cause or contribute to public health workers’ adverse mental health status*** might improve mental health outcomes during this and other public health emergencies.

The overall prevalence of symptoms of mental health conditions among public health workers was higher than previously reported in the general population (approximately 40.9%) (*1*). Prevalences of symptoms of depression and anxiety among public health workers were similar to those in previous reports among health care workers (*3*); however, prevalence of PTSD symptoms among public health workers was 10%–20% higher than that previously reported among health care workers (*2*), frontline personnel (*3*), and the general public (*1*). Symptoms of PTSD disproportionately affected public health workers who experienced work-related traumatic stressors (e.g., felt inadequately compensated or felt unappreciated at work), particularly those factors that affect workers’ personal lives (e.g., felt disconnected from family and friends because of workload). Traumatic and stressful work experiences related to the COVID-19 pandemic might have played a role in elevating the risk for experiencing symptoms of PTSD among public health workers.

Increases in adverse mental health symptoms among workers have been linked to increased absenteeism, high turnover, lower productivity, and lower morale, which could influence the effectiveness of public health organizations during emergencies (*8*,*9*). Among public health worker respondents, nearly 20% reported that their employer did not allow them to take time off; the inability to take time off had the largest impact on reporting symptoms of mental health. Approximately one quarter of public health workers did not know whether their workplace offered an employee assistance program. Even where available, employee assistance programs were not commonly accessed. Several strategies could reduce adverse mental health symptoms among public health workers during public health emergencies. For example, expanding staffing size (e.g., recruiting surge personnel to backfill positions) and implementing flexible schedules might reduce the need for long work hours; encouraging workers to take regular breaks and time off could help avoid overwork and reduce the risk for adverse mental health outcomes. In addition, implementing, evaluating, and promoting use of employee assistance programs could improve employee resiliency and coping.

The findings in this report are subject to at least four limitations. First, the study used a nonprobability–based convenience sample of public health worker respondents, and a completion rate could not be determined. Although the participating national public health membership associations reach many public health workers, the findings might not be representative of all state, tribal, local, and territorial public health workers in the United States. Second, self-reported mental health symptoms were assessed using screening instruments, which does not constitute clinical diagnosis of a mental health disorder; however, the screening instruments have been clinically validated (*5*–*7*). Third, participants were surveyed about symptoms experienced in the 2 weeks preceding the survey, which might not reflect all symptoms experienced during the pandemic. Finally, not all traumatic stressors or events experienced by public health workers were assessed by the survey, such as non–COVID-19 illnesses or financial insecurity.

During the COVID-19 pandemic, public health workers have experienced symptoms of depression, anxiety, PTSD, and suicidal ideation. Addressing work practices that contribute to stress and trauma is critical to managing workers’ adverse mental health status during emergency responses. Furthermore, strengthening work systems to encourage behavior changes that promote mental health, such as building awareness of symptoms of mental health conditions and developing sustainable coping strategies, might improve mental health conditions, particularly for public health workers who are at increased risk, including those who are younger (*10*) or transgender or nonbinary persons. In addition, employee assistance programs could be evaluated and adjusted to be more accessible and acceptable to workers and focus more on building workplace cultures that promote wellness and destigmatize requests for mental health assistance.

**Summary**

**What is already known about this topic?**

Increases in mental health conditions have been documented among the general population and health care workers during the COVID-19 pandemic; however, data on public health workers are limited.

**What is added by this report?**

Among 26,174 surveyed state, tribal, local, and territorial public health workers, 52.8% reported symptoms of at least one mental health condition in the past 2 weeks. Symptoms were more prevalent among those who were unable to take time off or worked ≥41 hours per week.

**What are the implications for public health practice?**

Implementing prevention and control practices that eliminate, reduce, and manage factors that cause or contribute to public health workers’ poor mental health might improve mental health outcomes during emergencies.

## Footnotes

* Respondents who did not report working at a state, tribal, local, or territorial public health agency or department in 2020 were excluded from the analysis.

† Membership associations that participated were the Association of Public Health Laboratories (APHL), the Association of State and Territorial Health Officials (ASTHO), the Council of State and Territorial Epidemiologists (CSTE), and the National Association of County and City Health Officials (NACCHO).

§ Respondents were asked if they had experienced specific traumatic events or stressors since March 2020, when COVID-19 was declared a pandemic; choices were yes/no/skip question.

¶ Symptoms of depression, anxiety, and post-traumatic stress disorder were scored and categorized by severity according to thresholds established by these validated tools. Those who scored ≥10.0 out of 27 on the PHQ-9 for depression, ≥3.0 out of 6 on the GAD-2 for anxiety, or ≥1.75 out of 4 on the IES-6 for PTSD were considered symptomatic for the respective conditions.

** Respondents who indicated that they would be better off dead or thought of hurting themselves at any time in the past 2 weeks were categorized as experiencing suicidal ideation.

†† Mental health outcome counts might not sum to total number of respondents because of missing data; counts for each category are those who answered all validated survey questions for that outcome.

§§ 45 C.F.R. part 46.102(l)(2), 21 C.F.R. part 56; 42 U.S.C. Sect. 241(d); 5 U.S.C. Sect. 552a; 44 U.S.C. Sect. 3501 et seq.

¶¶
https://www.cdc.gov/niosh/twh/guidelines.html

***
https://unhealthywork.org/category/mental-health-outcomes/
